# Supplementary figures and images for: Cryptococcus neoformans Recovered From Olive Trees (Olea europaea) in Turkey Reveal Allopatry With African and South American Lineages
Source: Front Cell Infect Microbiol. 2019 Nov 8;9:384. doi: 10.3389/fcimb.2019.00384 (PMC6856141; doi:10.3389/fcimb.2019.00384)

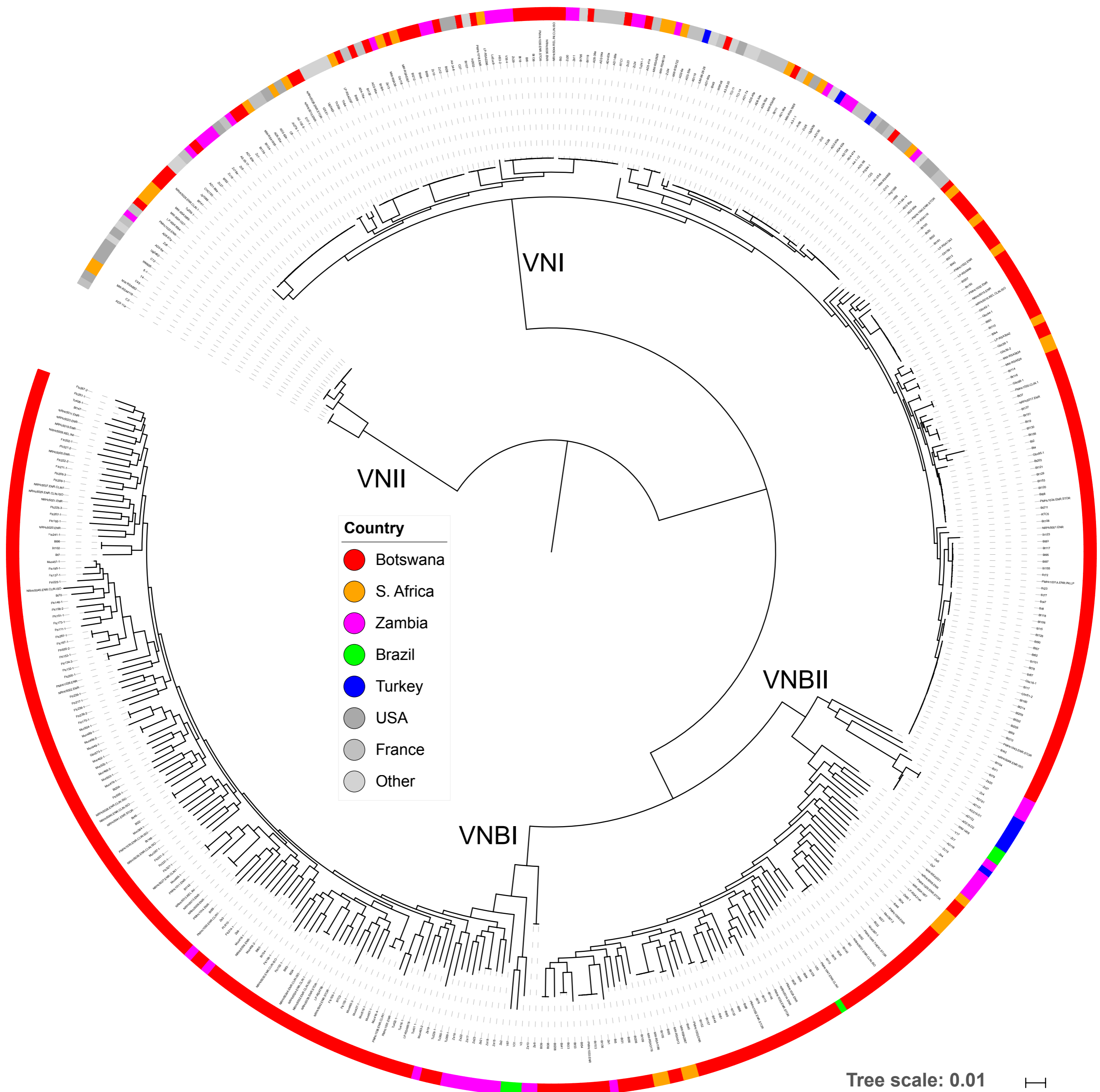

Supplement: Supplementary file 1 [file Data_Sheet_1.PDF]

## Slide 1
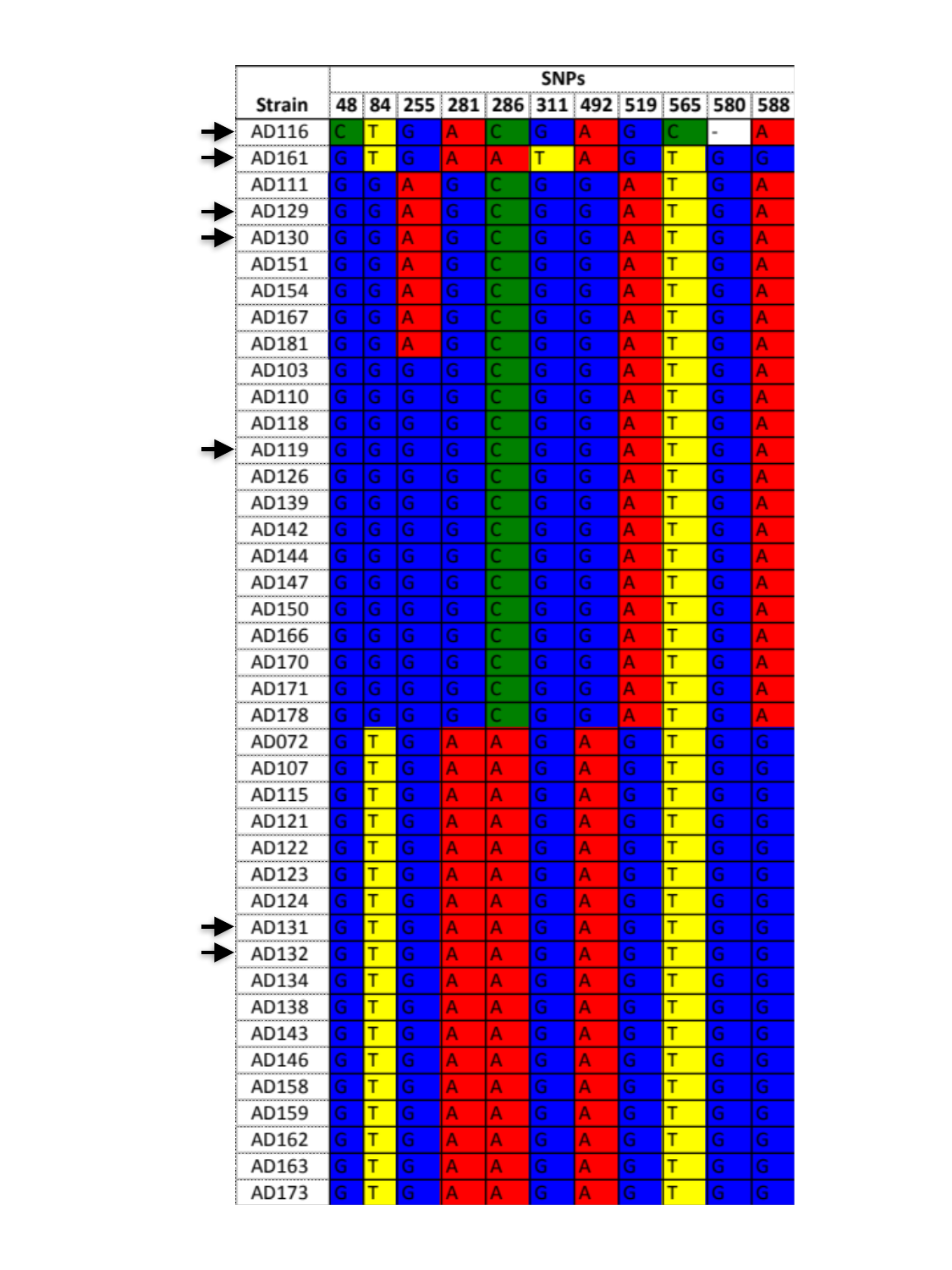

Supplement: Figure S1 — Genotyping of the URA5 locus revealed that the isolates from Turkey are genetically diverse. [file Presentation_1.PPTX]
