# Supplementary material for: Cryptococcus neoformans Recovered From Olive Trees (Olea europaea) in Turkey Reveal Allopatry With African and South American Lineages
Source: Front Cell Infect Microbiol. 2019 Nov 8;9:384. doi: 10.3389/fcimb.2019.00384 (PMC6856141; doi:10.3389/fcimb.2019.00384)
Supplement: Table S1 — Cryptococcus isolates recovered and analyzed in this study. [file Table_1.doc]

**Table S1. Primers used in this study.**

| Target | Primer symbol | Sequence (5′–3′) |
| --- | --- | --- |
| ITS | | |
|  | ITS1  ITS4 | TCCGTAGGTGAACCTGCGG  TCCTCCGCTTATTGATATGC |
| Mating-type alleles | | |
| *C*. *neoformans* | | |
|  | *STE20* A**a** F  *STE20* A**a** R  *STE20* Aα F  *STE20* Aα R | CTAACTCTACTACACCTCACGGCA  CGCACTGCAAAATAGATAAGTCTG  GGCTGCAATCACAGCACCTTAC  CTTCATGACATCACTCCCCTAT |
| *C*. *deneoformans* | | |
|  | *STE20* D**a** F  *STE20* D**a** R  *STE20* Dα F  *STE20* Dα R | CACATCTCAGATGCCATTTTACCA  AGCTCTAAGTCATATGGGTTATAT  CTTAATTCACAGCACCAGCCTA  GGTCATCACAGTCAGTCACCAC |

F, forward; R, reverse.
